# Predicted roles of long non-coding RNAs in abiotic stress tolerance responses of plants

**DOI:** 10.1186/s43897-024-00094-3

**Published:** 2024-05-15

**Authors:** IUH Imaduwage, Madhavi Hewadikaram

**Affiliations:** Department of Biomedical Sciences, Faculty of Science, NSBM Green University, Pitipana, Homagama, Sri Lanka

**Keywords:** Abiotic stress, Chemical treatment, Defense response, Long non-coding RNA, Non-coding RNA, Plant stress

## Abstract

The plant genome exhibits a significant amount of transcriptional activity, with most of the resulting transcripts lacking protein-coding potential. Non-coding RNAs play a pivotal role in the development and regulatory processes in plants. Long non-coding RNAs (lncRNAs), which exceed 200 nucleotides, may play a significant role in enhancing plant resilience to various abiotic stresses, such as excessive heat, drought, cold, and salinity. In addition, the exogenous application of chemicals, such as abscisic acid and salicylic acid, can augment plant defense responses against abiotic stress. While how lncRNAs play a role in abiotic stress tolerance is relatively well-studied in model plants, this review provides a comprehensive overview of the current understanding of this function in horticultural crop plants. It also delves into the potential role of lncRNAs in chemical priming of plants in order to acquire abiotic stress tolerance, although many limitations exist in proving lncRNA functionality under such conditions.

## Introduction

**Fig. 1:**
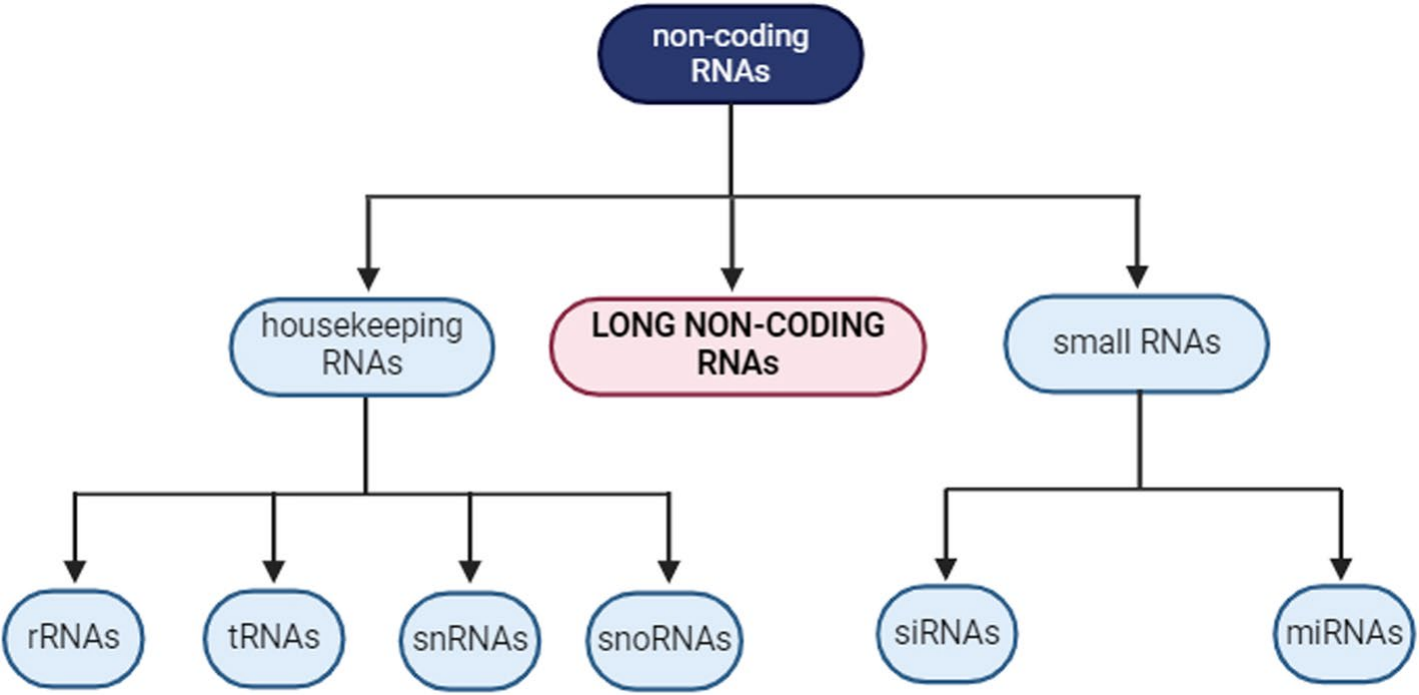
Classification of ncRNAs. Housekeeping RNAs have been extensively studied because of the fundamental nature of their expression. Other ncRNAs can be synthesized because of external stimuli and may be classified according to length into small RNAs (20–24 nucleotides) and long non-coding RNAs (≥200 nucleotides)

The eukaryotic genome is extensively transcribed into RNA. However, approximately 98% of the transcribed RNAs do not produce any functional proteins, and these RNAs are called non-coding RNAs (ncRNAs) (Wang et al. 2017). Housekeeping RNAs such as ribosomal RNAs, transfer RNAs, small nuclear RNAs, and small nucleolar RNAs are ncRNAs that have been comprehensively studied as they are fundamentally expressed and play a vital role in cell viability (Morey & Avner 2004). However, other ncRNAs are synthesized in response to external stimuli or during specific developmental stages (Fig. 1) (Szymański 2003).

**Fig. 2:**
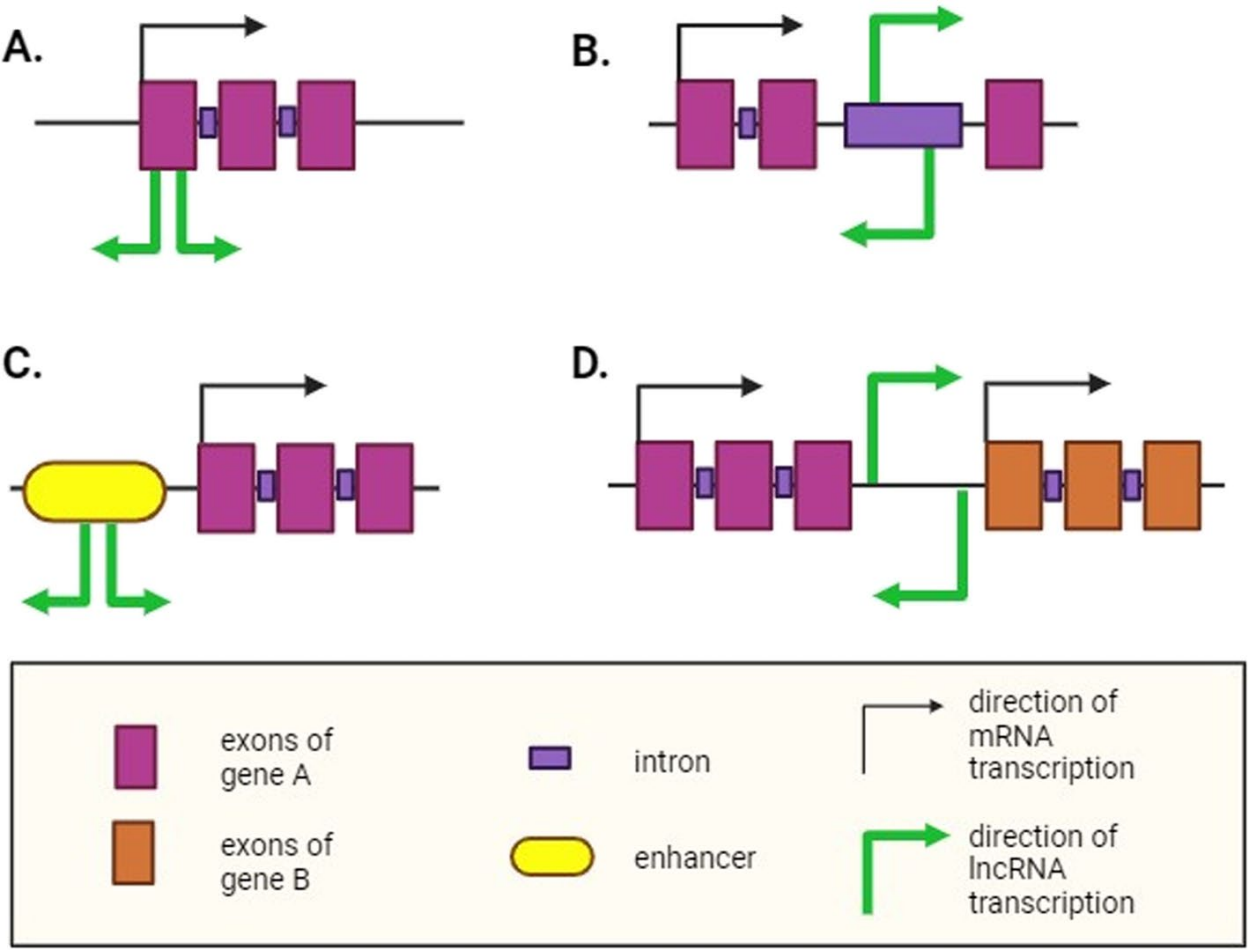
Classification of lncRNAs. Based on the genomic locations from which RNAs are transcribed relative to protein-coding regions, long non-coding RNAs (lncRNAs) can be classified into five distinct groups. **A** Exonic lncRNAs can partially or completely overlap with the exons of genes that code for proteins. They are transcribed in either the same direction (sense lncRNA) or the opposite direction (antisense lncRNA) as the mRNA. **B** Intronic lncRNAs originate from long introns of protein-coding genes and may be transcribed in either the same or the opposite direction as the mRNA. **C** Enhancer lncRNAs are transcribed from short enhancer regions of DNA. They may be bidirectional (as shown in the figure) or unidirectional, depending on their transcription direction. **D** Long intergenic non-coding RNAs (lincRNAs) arise from the intergenic region between two protein-coding genes and may be transcribed in either the same or the opposite direction of the nearest genes

The ncRNAs can be categorized according to their length. Small RNAs (sRNAs) comprising 20–24 nucleotides include small interfering RNAs (siRNAs) and microRNAs (miRNAs) (Axtell 2013). Long non-coding RNAs (lncRNAs) are typically described as having a length greater than 200 nucleotides. However, this value is arbitrary. A better way to describe lncRNAs is as RNAs that have functions distinct from protein-coding potential and have biogenesis mechanisms other than molecular scale-based cleaving or trimming, which are similar to mechanisms in sRNAs (Wierzbicki et al. 2021). LncRNAs may be classified in numerous ways according to their lengths, locations of protein-coding genes, biogenesis pathways, subcellular locations, functions, and so forth. One of the most common systems of classification is based on genomic origins (Fig. 2) (Wang & Chekanova 2017).

Despite being classified as “non-coding”, lncRNAs may have regions of under 100 codons known as small open reading frames that can be translated into biologically useful microproteins or peptides (Fesenko et al. 2021). One study found that these lncRNA-encoded peptides helped control the growth and differentiation of moss (*Physcomitrella patens*) (Fesenko et al. 2019). LncRNAs may consequently perform both protein-coding and non-protein-coding functions (Li & Liu 2019). The presence of lncRNAs is not proof of their functionality, and early research hypothesized that lncRNAs were simply byproducts of RNA processing or “transcriptional noise”. However, recent studies have revealed their roles in diverse biological processes in both plants and animals (Chekanova 2015). For example, dysregulation of these RNAs in mammals can impair normal cellular function, resulting in growth abnormalities and various diseases such as cancer (Weng et al. 2020). The functions of mammalian lncRNAs in immune responses, homeostasis, growth hormone production, organ development, and synaptic function have also been comprehensively studied (Dhanoa et al. 2018; Mattick et al. 2023; Wang & Chang 2011). In plants, lncRNAs play a role in controlling epigenetics, flowering time, organogenesis, and photomorphogenesis, and they also control gene expression to help plants deal with stress (Bardou et al. 2014; Berry & Dean 2015; Matzke & Mosher 2014; Wang et al. 2014).

Two categories of external conditions that disrupt plant growth, development, and productivity are biotic and abiotic stresses (Gull and Ahmad Lone 2019). Living organisms such as bacteria and fungi are the root causes of biotic stress, while environmental factors such as heat, drought, salinity, etc. cause abiotic stress (Rodríguez et al. 2005). Horticultural crops, including fruits, vegetables, and medicinal plants, are vital to human health and the global economy. As plants are immobile, they need to acclimatize to adverse conditions in order to survive (Patra et al. 2016). LncRNAs may play a role in plant defense mechanisms against abiotic stress.

As abiotic stress severely restricts crop yield and productivity, several strategies to enhance plant stress tolerance have been explored. The exogenous application of compounds such as methyl jasmonate and salicylic acid (SA) is one of the most efficient of these strategies (Hosseinifard et al. 2022). This review summarizes the research status of the effects of several abiotic stresses on lncRNA expression in different horticultural plant species. It also examines the small body of literature regarding the possible link between lncRNAs and exogenous chemical treatments in plant defense against abiotic stress.

### Functional mechanisms of lncRNAs

**Fig. 3:**
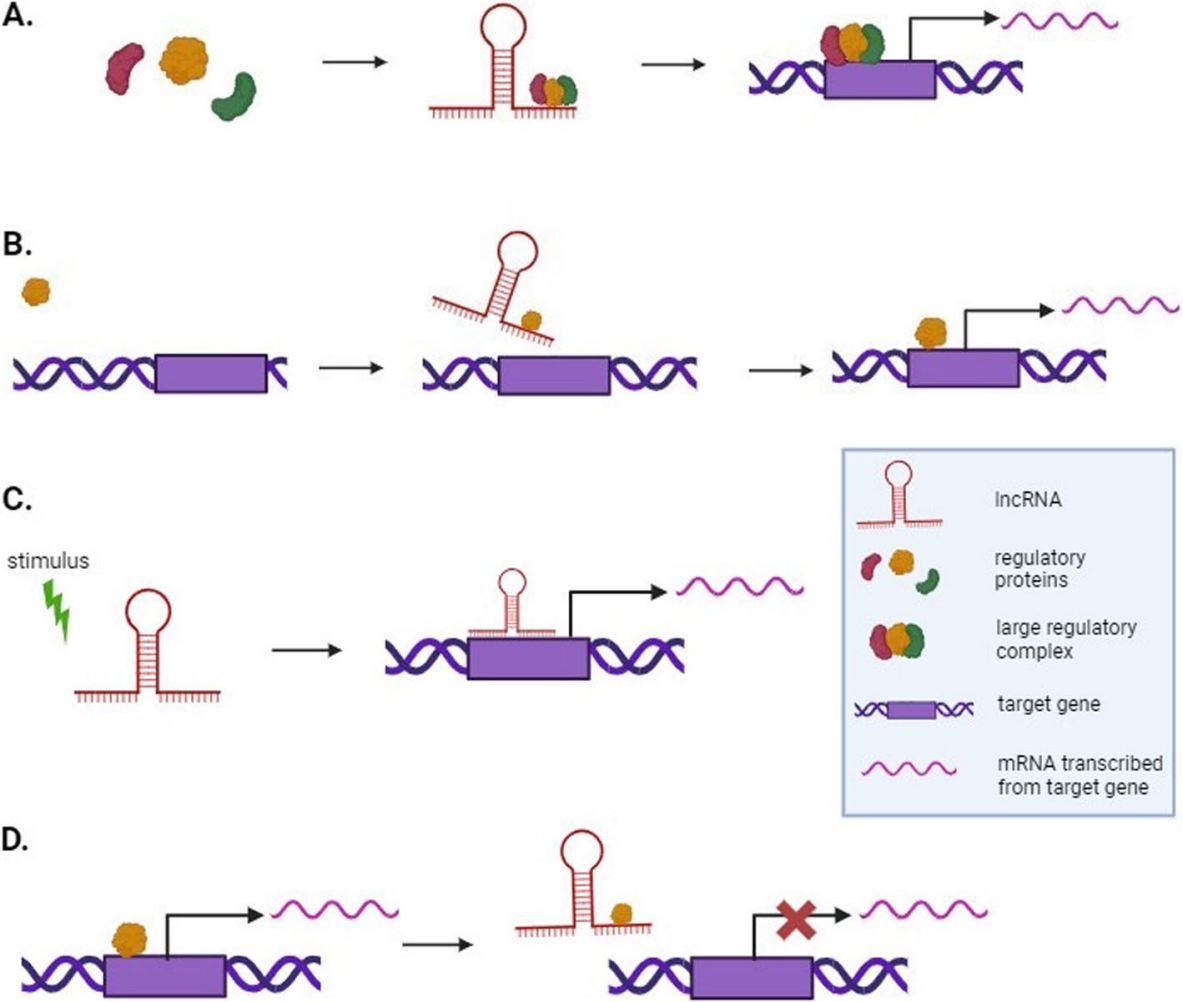
Mechanisms of action of lncRNAs. LncRNAs may regulate gene expression by acting as scaffolds, guides, signals, or decoys. **A** Scaffolds bind to multiple small molecular components simultaneously and act as a platform for assembling these components into large regulatory complexes that activate or inhibit gene expression. **B** Guides bind to specific regulatory proteins and direct their localization to specific target genomic loci where gene expression is regulated. **C** Signals function as molecular cues because they precisely start transcription at certain times and places, letting cells respond to different stimuli. They function in various ways, such as by directly binding to the target site to perform a regulatory role. **D** Decoys act by binding to RNA-binding proteins, such as transcription factors or chromatin modifiers, and sequester them away from their intended targets, thus inhibiting these proteins from performing their roles. Note: The regulatory proteins are shown as positively regulating gene expression, but they may also inhibit gene expression

A growing number of lncRNAs have been reported in plant transcriptomes during the last few decades, although only a small percentage of these have been functionally characterized. Gene expression is controlled at the epigenetic, transcriptional, post-transcriptional, translational, and post-translational levels in eukaryotic cells. LncRNAs play a major role in the regulation of gene expression on many of these levels (Statello et al. 2021). They can perform their regulatory roles in various ways by acting as scaffolds, guides, signals, or decoys (Fig. 3) (Chowdhary et al. 2021). Certain lncRNAs can perform more than one of these roles; therefore, it is difficult to categorize them into a single group (Wang & Chang 2011).

### Scaffolds

LncRNAs interact with proteins by complementary sequence motifs or secondary or tertiary structures. They help assemble and connect small protein complexes to form large functional units (Blythe et al. 2016; Ma et al. 2022). For example, the responsive lncRNAs *TCONS_00202587* and *TCONS_00260893* controlled their targets by acting as RNA scaffolds. This helped protect and recover photosynthetic processes, stopped membrane peroxidation, and lowered DNA damage in poplar (*Populus simonii*) under heat stress (Song et al. 2020).

### Guides

Guide lncRNAs attach to molecules such as transcriptional co-regulators or chromatin regulatory protein complexes and move them to specific parts of the genome where target gene expression is controlled (Lv et al. 2023; Ma et al. 2022). In the first intronic region of *FLOWERING LOCUS C* (*FLC*), *COLD-ASSISTED INTRONIC NON-CODING RNA* (*COLDAIR*) recruits polycomb repressive complex 2, which results in the H3K27me3 alteration of histones during the early vernalization of plants (Heo & Sung 2011).

### Signals

LncRNAs can connect to DNA-binding proteins such as transcription factors (TFs) and histone-modifying complexes. This can change the levels of transcription, splicing, and translation to control gene expression. Thus, they act as indicators of transcriptional activity at a particular time and space (Wang & Chang 2011).

TFs contain DNA-binding domains that bind to specific regions of genes and control the rate of transcription, regulating the rate and timing of gene expression. In plants, lncRNAs may directly interact with TFs or indirectly interact with the multiprotein complex “mediator”, which in turn interacts with TFs (Yang et al. 2023; Yin & Wang 2014). An example of the first type is how the lncRNA *AUXIN-REGULATED PROMOTER LOOP* (*APOLO*) and the TF WRKY42 work together in *Arabidopsis thaliana* under cold stress. A complex called WRKY42–*APOLO* changes the 3D conformation of the chromatin at the *ROOT HAIR DEFECTIVE 6* (*RHD6*) genomic region. This turns on the *RHD6* region and causes *ROOT HAIR DEFECTIVE 6-LIKE 2* (*RSL2*) and *RSL4* to be expressed. The complex also binds to *EXTENSIN 3*. Both actions result in root hair cell elongation (Moison et al. 2021; Pacheco et al. 2021). In *Arabidopsis*, *ELF18-induced long non-coding RNA1* (*ELENA1*) binds to a mediator subunit called MED19a and acts on the promoter region of *PATHOGENESIS-RELATED GENE 1* (*PR1*) to protect the plant from *Pseudomonas syringae*. It has been proposed that ELENA1 recruits MED19a to *PR1* by interacting with another mediator, MED26b (Seo et al. 2017).

LncRNAs can directly regulate transcription as well (Yang et al. 2023). In response to lead (Pb^2+^) toxicity, 226 lncRNAs, including *PMAT* and *PtoMYB46*, were induced and differentially expressed in poplar (*P. tomentosa*). *PMAT*, or *PtoMYB46*, regulated by *PMAT*, stops *PtoMATE* transcription and thus its translation, causing cells to produce less citric acid and take in more Pb^2+^ (Chen et al. 2022).

Protein trafficking, a process critical to plant stress response, refers to the movement of proteins within cells from one subcellular compartment to another (Droujinine et al. 2021). LncRNAs can mediate this process. For example, nuclear-localized RNA binding protein 1 was re-localized to the cytoplasm by the lncRNA *ENOD40* in *Medicago truncatula* (Yang et al. 2023).

LncRNAs can also stop certain proteins from interacting with each other. This stops the formation of certain macromolecular complexes that are needed to control gene expression (Yang et al. 2023). This was observed in the early stages of endosperm development in rice (*Oryza sativa*), where a helicase family protein (HeFP) controls how tubulin works. *MISSEN*, a lncRNA, competitively inhibits the interaction between HeFP and tubulin, leading to abnormal cytoskeletal polymerization and the formation of slightly larger seeds (Zhou et al. 2021). During biotic stresses in *Arabidopsis*, *ELENA1* interacts with and frees FIBRILLARIN 2, a negative transcriptional regulator, from the *PR1* promoter. This makes room for the positive regulator MED19a to bind to it (Seo et al. 2019).

In chromatin remodeling, chromatin is rearranged to make it accessible to TFs or other DNA-binding proteins in order to regulate gene expression (Jiang et al. 2023). Post-transcriptional modifications of histone proteins that activate or repress transcription in this fashion can be regulated by lncRNAs, which recruit chromatin-modifying complexes to perform the function. The activation of histone modifications can also be moderated by lncRNAs (Yang et al. 2023).

Regulatory DNA sequences can make physical contact with target genes by chromatin looping to control transcription over long distances (Holwerda & De Laat 2012). By bringing in chromatin-modifying complexes, lncRNAs may be involved in the formation of chromatin loops between lncRNA and its target (Yang et al. 2023). For example, when *Arabidopsis* is exposed to exogenous abscisic acid (ABA), the *MARneral Silencing* (*MARS*) lncRNA causes the chromatin loop to form R-loops (Roulé et al. 2022).

R-loops are made up of an RNA–DNA duplex and an unpaired DNA strand. They can control gene expression at the chromosomal ends (Belotserkovskii et al. 2018). The involvement of plant lncRNAs in R-loop synthesis has been proven. The *APOLO* lncRNA creates an R-loop that controls the activity of genes in *Arabidopsis* that react to distal auxin as the lateral roots expand (Ariel et al. 2020).

Alternative splicing alters pre-mRNA structures before translation. During this process, exons from the same gene are linked in different ways to form different but related mRNA transcripts (Greenberg et al. 2013). The mechanism of alternative splicing can be influenced by lncRNAs, either by binding to spliceosome components or nuclear speckle RNA-binding proteins (Yang et al. 2023). *Flowering-associated intergenic lncRNA* (*FLAIL*) in *Arabidopsis* interacts with components of the spliceosome to influence the expression of target mRNA. Flowering is repressed by *FLAIL* by regulating alternative splicing (Jin et al. 2023).

LncRNAs can also regulate gene expression by inhibiting or enhancing the association between mRNA transcripts and polysomes (Yang et al. 2023). There is a nitrogen-fixing symbiotic relationship between *M. truncatula* and *Sinorhizobium meliloti*. The association between an alternative variant of the lncRNA *trans-acting small interference RNA3* (*ALT TAS3*) and polysomes is enhanced in response to rhizobia, increasing nodule development and therefore increasing the chances of productive symbiosis (Traubenik et al. 2020).

### Decoys

Transcriptional regulators are prevented from binding to their binding sites by decoy lncRNAs to regulate gene expression. miRNAs are used by the RNA-induced silencing complex in gene silencing. In plants, certain lncRNAs with miRNA recognition regions that are similar to miRNA targets can act like miRNA targets to stop miRNA activity by binding to miRNAs and stopping them from interacting with the actual targets (Dupon et al. 2009). These lncRNAs are known as competing endogenous RNAs (ceRNAs) or lncRNA sponges, and they play a significant role in reducing gene expression (Wang & Chekanova 2017; Yang et al. 2023). In wheat (*Triticum aestivum*), 849 lncRNAs changed their expression when the plant was exposed to alkaline stress and were decoys for 115 conserved miRNAs (Wei et al. 2022). Many lncRNAs also act as precursors of shorter regulatory RNAs, such as miRNAs or siRNAs, particularly those that actively participate in the RNAi pathway. In cotton (*Gossypium hirsutum*), 88 lncRNAs were precursors of 57 miRNAs (Hamid et al. 2020). These mechanisms modulate many developmental processes in plants, particularly those related to reproduction, like flowering. They are also key to plant responses to external stresses.

### Stress tolerance responses in plants

The effects of different types of stresses on lncRNAs in the model plant *A. thaliana* have been extensively studied. For example, *cold-induced long antisense intragenic RNA* (*COOLAIR*), *COLD ASSISTED INTRONIC NON-CODING RNA* (*COLDAIR*), and *COLD OF WINTER-INDUCED NON-CODING RNA FROM THE PROMOTER* (*COLDWRAP*) found in *Arabidopsis* and other *Brassicaceae* plants are among some of the best-characterized lncRNAs responsive to a stress condition, although they are not involved in stress tolerance. Vernalization is a process in which prolonged exposure to cold stress ultimately promotes flowering. *FLC* encodes a TF that negatively regulates flowering, and the lncRNAs in question assist in downregulating *FLC* expression under cold stress. *COOLAIR* can repress *FLC* transcription by directly binding to the locus and modifying chromatin or by forming an R-loop. *COLDWRAP* can form a chromatin loop with *COLDAIR*, which has a similar repressive function. *COLDAIR* also acts as a guide, as mentioned above. These RNAs can use several of the previously stated mechanisms of action to perform their roles (Crevillén et al. 2013; Heo & Sung 2011; Kim & Sung 2021; Xu et al. 2021)

Twelve lncRNAs were differentially expressed in the two *A. thaliana* ecotypes, *Columbia* (Col) and Landsberg *erecta* (L*er*), lacking phosphate, which is a nutrient deficiency stress. One hypothesis is that certain lncRNAs are expressed in the genomes of Col and L*er* in connection with known regulators of the phosphate-starvation response. For example, the high expression of phosphate transporters in L*er* may cause the cell to take in more phosphate (Blein et al. 2020). Phosphate deficiency in *Arabidopsis* can also cause lncRNAs to activate the RNA-directed DNA methylation silencing pathway. This is a plant-specific regulatory system in which ncRNA molecules direct the methylation of specific DNA sequences (De Oliveira Urquiaga et al. 2021; Erdmann & Picard 2020; Yong-Villalobos et al. 2015).

It is of utmost importance to study the mechanisms of stress responses in horticultural crops. Abiotic stress conditions restrict the locations where plants may be grown and can have a significant negative impact on global agricultural productivity. There is an unambiguous relationship between escalating climate change and reduced agricultural productivity, making abiotic stress-induced losses in crop yield inevitable in the coming years. With a rapidly increasing world population, several preventative and acclimatization strategies must be used in intervention plans to maintain agricultural sustainability and prevent financial losses (Fawzy et al. 2020).

Studying lncRNAs in plants under stress can reveal unknown regulatory mechanisms involved in stress response and adaptation. Understanding these mechanisms can help identify potential targets for genetic engineering to enhance stress tolerance in crops and improve their productivity under adverse environmental conditions. In addition, studying the expression patterns of lncRNAs under stress can help identify potential biomarkers that can be used to monitor stress responses and predict plant performance under different stress conditions.

To date, several studies have attempted to examine the effects of abiotic stresses on lncRNAs in horticultural crops (Table 1).

**Table 1:** Recent studies on lncRNAs in relation to plant stress (2019–2024)

	**Plant Species**	**No. of Differentially Expressed LncRNAs**	**Stress**	**Reference**
1	*Capsicum annuum* (bell pepper)	1887	osmotic	(Baruah et al. 2021)
		2069	salt	
		2101	cold	
		2833	heat	
2	*Triticum aestivum* (bread wheat)	1515	drought	(Li et al. 2022)
3	*Camellia sinensis* (tea)	172	salt	(Wan et al. 2020)
4	*Brassica juncea* (Indian mustard)	1614	heat and drought	(Bhatia et al. 2020)
5	*Manihot esculenta* (cassava)	117	drought	(Ding et al. 2019)
6	*Melilotus albus* (honey clover)	550	salt	(Zong et al. 2021)
7	*B. napus* (rapeseed) Q2 genotype	126	drought	(Tan et al. 2020)
	*B. napus* (rapeseed) Qinyou 8 genotype	359		
8	*B. rapa* (Chinese cabbage)	93	heat	(Eom et al. 2021)
9	*Oryza sativa* (rice) ssp. *japonica*	97	heat	(Zhang et al. 2021)
	*O. sativa* (rice) ssp. *indica*	103		
10	*Panicum virgatum* (switchgrass)	368	drought	(Guan et al. 2024)
11	*Olea europaea* (olive)	2076	heavy metal (Al)	(Wu et al. 2023)
12	*Hordeum vulgare* (barley)	195	heavy metal (Cd)	(Zhou et al. 2023)
13	*Populus trichocarpa* (black cottonwood)	1183	salt	(Ye et al. 2022)
14	*Solanum pennellii* (wild tomato)	137	salt	(Li et al. 2022)
	*S. lycopersicum* (cultivated tomato M82)	154		

### Salt stress

**Fig. 4:**
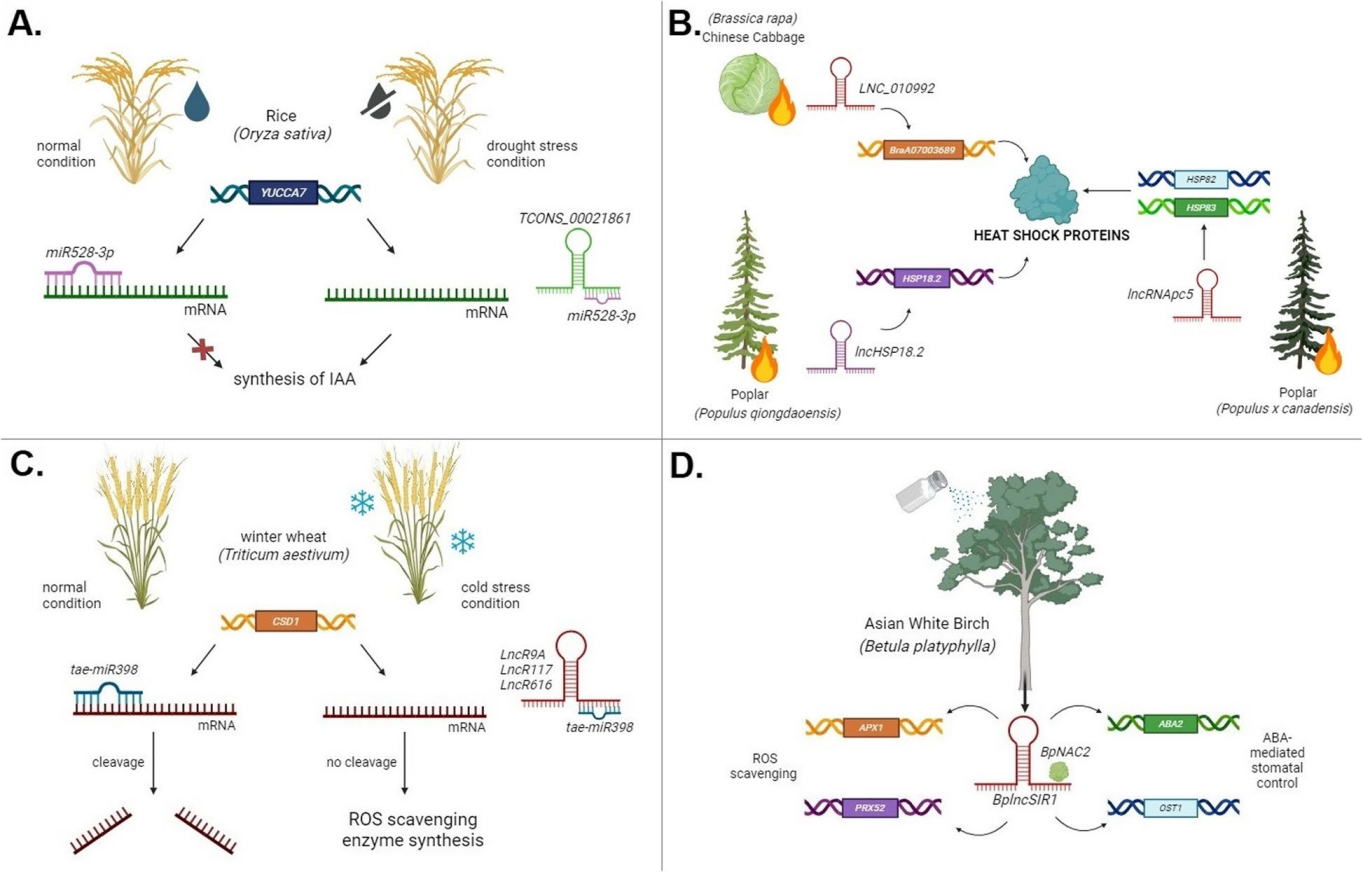
Studies that investigated the role of lncRNAs in enhancing plant tolerance to abiotic stress. **A** The lncRNA *TCONS_00021861* acts by sponging *miR528-3p* and preventing its binding to target mRNA, leading to increased levels of the plant growth regulator IAA to counteract the effects of drought stress in rice (*Oryza sativa*). **B** LncRNAs induced in *Brassica rapa*, *Populus qiongdaoensis*, and *Populus* × *canadensis* under heat stress regulate genes involved in the production of heat-shock proteins, which can restore misfolded proteins. **C** LncRNAs in wheat (*Triticum aestivum*) under cold stress act as ceRNAs by competing with tae-miR398 to prevent the cleavage of target mRNA, leading to the synthesis of ROS-scavenging enzymes. **D** The lncRNA *BplncSIR1* enhances salt stress tolerance in Asian white birch (*Betula platyphylla*). *BplncSIR1* binds to the transcription factor *BpNAC2*, activating genes involved in ABA-mediated stomatal control and the production of ROS-scavenging enzymes

A study on Asian white birch (*Betula platyphylla*) focused on a specific lncRNA, *BplncSIR1*, which was differentially expressed under salt stress. Transgenic lines of this plant with overexpression and inactivation of *BplncSIR1* were generated to observe the phenotypic consequences of altering the lncRNA being studied. There was a positive correlation between high expression levels of *BplncSIR1* and accelerated plant growth, reduced water loss due to reduced stomatal aperture width, decreased reactive oxygen species (ROS) accumulation, as well as elevated activity of antioxidant enzymes under conditions of increased salinity. The lncRNA was proposed to function by binding to and regulating the expression of the TF *BpNAC2*, which in turn activated genes such as *ascorbate peroxidase 1* (*APX1*), *peroxidase 52* (*PRX52*), *abscisic acid-deficient 2* (*ABA2*), and *open stomata 1* (*OST1*). Both *ABA2* and *OST1* are key genes in ABA-mediated stomatal control, while *APX1* and *PRX52* code for ROS-scavenging enzymes (Fig. 4). Notably, the authors of this study confirmed that these functions of *BplncSIR1* in Asian white birch were not due to the short peptide that it encodes (Jia et al. 2023).

On exposure to salt stress, 126 and 133 lncRNAs were found in the M-81E and Roma lines of sweet sorghum (*Sorghum bicolor*), respectively. These lncRNAs were found to potentially control transcription by competing with miRNAs to bind to target mRNAs. For instance, *lncRNA13472* and *sbi-MIR169b-p3* might compete to bind to *SORBI_3010G218400*. This gene codes for a V-type proton ATPase subunit that is associated with membrane transport. The ceRNA network in this plant may also affect other target genes that code for proton pumps, transporters, enzymes, and TFs (Sun et al. 2020).

Chickpea (*Cicer arietinum*) exposed to salt stress resulted in the differential expression of 3450 lncRNAs. Functional annotation suggested that lncRNAs control salt tolerance by changing the levels of several TFs, potassium transporters, serine/threonine protein kinases, and aquaporins, as well as methylation pathways. TFs belonging to families such as WRKY, NAC, and ERF play a prominent role in stress tolerance. A total of 80 distinct lncRNAs were predicted to interact with 136 different miRNAs as endogenous target mimics of miRNAs. The study suggests that these mimics change the expression of the penta-tricopeptide repeat gene family. This could strengthen the plant’s defense by inhibiting the stomatal opening. Simple sequence repeats, which are vital molecular markers, were also reported in 614 lncRNAs. The authors of this paper suggested that leveraging lncRNA sequences was crucial to develop lncRNA-related markers for crop improvement (Kumar et al. 2021).

### Heat stress

In Chinese cabbage (*Brassica rapa*), 1229 differentially expressed lncRNAs were identified as being responsive to heat stress, with the number expressed increasing gradually with the increase in heating time. Using functional enrichment analyses, lncRNAs were predicted to control the activity of heat-shock proteins (*HSP*s) and genes related to HSPs. For example, *LNC_010992* was thought to regulate *BraA07003689*. High temperatures can cause protein misfolding, and *HSP*s play a pivotal role in the restoration of functional folding in heat stress-damaged proteins. Other genes whose expression was regulated in heat-treated Chinese cabbage were those related to the protein ubiquitin system, such as *BraA01004433*, which may assist in the degradation of denatured proteins. In addition, *LNC_013535* regulated the expression of *BraA09001034*, a gene encoding dehydrin. Dehydrins are typically involved in protecting a plant against dehydration that may be caused by heat. LncRNAs were also co-expressed with three *major latex protein-like* genes. These genes positively responded to heat stress and are considered part of the ABA-mediated stress tolerance pathway. The phytohormone ABA has been shown to alleviate the effects of heat stress (Chan 2012; Song et al. 2021). However, lncRNAs were also involved in the downregulation of *PYR/PYL* genes, despite their coding for ABA receptors. This indicates that gene expression is not a necessity for the ABA-mediated stress tolerance pathway. Finally, most miRNAs in the constructed ceRNA network were of the two types expressed in response to heat stress in other studies as well, although their exact function is unclear (Ahmed et al. 2019).

Poplar (*P. qiongdaoensis*) seedlings treated with heat stress showed 25 differentially expressed lncRNAs, one of which targeted six *HSP* genes. This lncRNA (*lncHSP18.2*) could *cis-*regulate the expression of the *HSP18.2* gene (Xu et al. 2020). Similarly, in another study where poplar (*Populus × canadensis*) trees underwent heat stress conditions, *lncRNAPc5* could target and regulate the expression of *HSP82* and *HSP83* (Fig. 4) (Xu et al. 2020).

In pear (*Pyrus* spp.), *HILinc1* induced by heat upregulated the mRNA of its target gene, *PbHILT1*, by complementary base pairing. *PbHILT1* then interacted with the transcriptional factor *PbHSFA1b*, which in turn enhanced the expression of an important heat shock response gene, *PbMBF1c* (Table 2). Plants overexpressing *HILinc1* were therefore highly thermotolerant in the presence of heat stress. This study is also significant because its methodology used several biochemical techniques to confirm the proposed mechanisms of action, unlike other studies (Zhang et al. 2022).

**Table 2:** Studies concerning specific lncRNAs that were predicted to enhance stress tolerance

	**Plant Species**	**Name of LncRNA**	**Stress**	**Reference**
1	*Manihot esculenta* (cassava)	*DIR* (*DROUGHT-INDUCED INTERGENIC lncRNA*)	drought	(Dong et al. 2022)
2	*M. esculenta* (cassava)	*CRIR1* (*cold-responsive intergenic lncRNA 1*)	cold	(Li et al. 2022)
3	*Gossypium hirsutum* (cotton)	*LncRNA973*	salt	(Zhang et al. 2019)
4	*Medicago truncatula* (barrelclover)	*MtCIR2*	cold	(Zhao et al. 2023)
5	*Pyrus* spp. (pear)	*HILinc1* (*heat-induced long intergenic non-coding RNA 1*)	heat	(Xu et al. 2020)

Jujube (*Ziziphus jujuba*) seedlings exposed to high temperatures for different periods expressed many unique differentially expressed lncRNAs at each time point, with only 40 lncRNAs being commonly expressed at all time points. Although the potential target genes of these lncRNAs were enriched in the pathways associated with response to heat stress, the study was unable to confirm any exact mechanisms (Hao et al. 2021).

In the leaves of cucumber (*Cucumis sativus*), 108 lncRNAs were differentially expressed following the application of heat stress. The lncRNAs *TCONS_00031790*, *TCONS_00014332*, *TCONS_00014717*, and *TCONS_00005674* were all predicted to competitively bind to *miR9748*. This miRNA was also targeted by mRNAs such as *Csa1M690240.1*, *Csa6M091930.1*, and *Csa7M405830.*1, which are key players in the hormone signal transduction pathway. *Csa1M690240.1* and *Csa7M405830.1* may specifically change the levels of indole-3-acetic acid (IAA) and ethylene in plants, both of which are synthesized less under heat stress conditions (He at al., 2020). *miR9748* has also been shown to affect HSP90 levels in plants (Cakir et al. 2016).

### Cold stress

In the leaves and roots of *M. truncatula*, 983 and 1288 lncRNAs, respectively, were responsive to cold stress. Interestingly, lncRNA distribution in the leaves and roots depicted clear locational preferences, suggesting that these lncRNAs are tissue specific. While several putative targets of the cold-responsive lncRNAs were predicted, one of the most significant was a tandem array of *CBF/DREB1* genes located in a crucial cold tolerance region on chromosome 6. These genes code for transcriptional activators that are directed at *CBF-targeted cold-regulated* genes, which may play a role in freezing tolerance (Zhao et al. 2020).

The winter wheat (*T. aestivum*) cultivar Dn1 is known for its resilience in low temperatures. An miRNA isolated from this plant, *tae-miR398*, typically cleaves the mRNA produced by the *CSD1* gene that codes for Cu/Zn superoxide dismutase (SOD), reducing its synthesis. SODs are ROS-scavenging enzymes; they eliminate ROS, which causes oxidative damage to plants. Under cold stress conditions, *tae-miR398* in Dn1 was downregulated, and thus, *CSD1* was upregulated. *LncR9A*, *lncR117*, and *lncR616* were capable of interacting with this miRNA. These lncRNAs might compete with *tae-miR398* to stop it from binding and cleaving target mRNA. This would increase SOD activity and protect the plant (Fig. 4) (Lu et al. 2020).

Grapevine (*Vitis vinifera*) subjected to cold stress had 813 differentially expressed lncRNAs. Some targets of these lncRNAs were genes that coded for CBF4 TF, late embryogenesis abundant (LEA) protein Lea14-A, and WRKY TF 41 (Wang et al. 2019). CBF, as mentioned earlier, as well as LEA and WRKY proteins, are vital in the freezing tolerance response of plants (Sasaki et al. 2014; Xiao et al. 2008; Zou et al. 2010). LncRNA expression may even be induced in the fruits of plants under stress conditions. For instance, 380 lncRNAs were differentially expressed in chilled bell pepper (*Capsicum annuum*) fruits. A ceRNA network that targeted the synthesis of key enzymes, including serine/threonine protein kinases and β-galactosidases, contained 81 of these lncRNAs (Zuo et al. 2018).

### Drought stress

In a comprehensive study where rice (*O. sativa*) was placed in a water deficit condition, 98 lncRNAs were differentially expressed. In the constructed ceRNA network, the *TCONS_00021861/miR528-3p/YUCCA7* triplet had the most significant positive correlation. The *YUCCA* gene family is responsible for IAA synthesis and thus subserves plant growth regulation. Drought-stressed rice showed upregulated *miR528-3p* expression and downregulated lncRNA *TCONS_00021861* and *YUCCA7* expression. The overexpression of *TCONS_00021861* implies that lncRNA positively regulated *YUCCA7* as IAA levels significantly increased. The opposite occurred with *miR528-3p* overexpression, implying that miRNA negatively regulated *YUCCA7*. Thus, *TCONS_00021861* could increase IAA levels in the plant by sponging *miR528-3p* and preventing its binding to *YUCCA7* (Fig. 4). As expected, the *TCONS_00021861* overexpression group showed increased weight and length in the plant leaves and roots due to the amplified expression of IAA.

In addition, abiotic stress tends to result in ROS accumulation within the plant. In rice *TCONS_00021861* overexpression lines, no increase in H_2_O_2_ and O_2_ contents was observed under drought stress. This contrasted with *miR528-3p* overexpression lines, where ROS content was significantly increased. Interestingly, the authors of this study also examined the ultrastructure of mesophyll cells of the plant. Compared with the control group, chloroplasts were damaged and granal stacking was disrupted, among other signs of damage to organelles in drought-stressed leaves. However, in the *TCONS_00021861* overexpression group, chloroplast damage was minimal and granal stacking was unaffected. This overall mitigation of stress-induced plant damage may be a result of elevated IAA signaling caused by *TCONS_00021861* lncRNA (Chen et al. 2021).

In beet (*Beta vulgaris*), 386 differentially expressed lncRNAs were induced under drought stress, with *TCONS_00055787* being upregulated by more than 6000-fold. Flavonoids are secondary metabolites known to exert a protective effect on plants affected by drought stress. The lncRNAs *TCONS_00009457* and *TCONS_00088109* targeted the genes *BVRB_1g007170* and *BVRB_1g016280*, respectively, both of which occur in the flavonoid biosynthetic pathway of plants. In addition, the lncRNAs *TCONS_00055970* and *TCONS_00056083* were predicted to target the *BVRB_6g151690* and *BVRB_6g152260* genes, which code for chalcone synthase, an enzyme necessary in flavonoid synthesis. Other lncRNAs targeted genes that encoded fructose-1,6-bisphosphatase and malate dehydrogenase, which are key players in photosynthetic processes. These lncRNAs might be involved in altering the photosynthetic processes that occur in beet under drought conditions (Zou et al. 2023).

Another study found 1395 drought-specific lncRNAs in tea (*Camellia sinensis*), many of which were predicted to act as target mimics of miRNAs. Analysis results showed that the initial target genes of these miRNAs were involved in pathways such as the citrate cycle, purine and thiamine metabolism, and the biosynthesis of unsaturated fatty acids. Exactly how lncRNAs perform their hypothesized function was not determined (Baruah et al. 2021).

Many of the proposed mechanisms of action discussed in the studies above have not been substantiated with evidence from biochemical experimentation. Computational prediction tools are an essential first step in characterizing newly reported lncRNAs. However, these molecular functions must be experimentally proven, a promising avenue for future research.

All these studies discuss the basal responses of plants to different stresses. However, in severe stress conditions, this response may not be sufficient to defend a plant against negative consequences.

## Priming-induced acquired stress tolerance

Priming-induced acquired stress tolerance is a phenomenon in which exposure to mild stress can help a plant cope with any ensuing stresses better, compared with a previously unexposed plant (Nair et al. 2022). Priming can lead to a more rapid and amplified stress tolerance response because of the plant “remembering” the previous stress exposure. The priming stimulus and the subsequent stress the plant is exposed to may be of the same type (*cis*-priming) or of different types (*trans*-priming or cross-stress tolerance) (Johnson & Puthur 2021). An example of the former is shown in a study in which chickpea (*C. arietinum* cv. Anuradha) and lentil (*Lens culinaris* cv. Ranjan) seeds primed with NaCl displayed improved tissue tolerance under salt stress conditions (Khemka et al. 2016). Contrarily, cold stress priming of Bermuda grass (*Cynodon dactylon)* led to increased tolerance to salt stress and is an example of the latter (Noor et al. 2023). Priming-induced plant stress memory ranges from short-term (a few hours) to long-term (several weeks) (Bäurle and Trindade 2020). Sometimes, priming-induced stress memory is passed down to plant offspring because of direct exposure of the parent plant to a stressor, known as “intergenerational stress memory”. Conversely, “transgenerational stress memory” is demonstrated when the effects of ancestor stressor exposure were present in the offspring generation even if the parent generation was not exposed to the stressor. For instance, stress memory of two parental generations of *wheat (T. aestivum*) enhanced the growth and survival of offspring in drought stress conditions (Kambona et al. 2023). A study that investigated short-term stress memory in rice (*O. sativa*) under drought stress found that memory-related expression patterns were observed in 6.33% of identified lncRNAs. One such lncRNA, *TCONS_00028567*, was predicted to be a precursor of *osa-MIR1428e* and may act as a post-transcriptional regulator of *serine/threonine protein kinase 10* gene products, which are key in the ABA signaling pathway that plays a role in drought stress tolerance (Li et al. 2019).

## Link between lncRNAs and exogenous chemical application in plants

The exogenous application of certain chemicals that may mimic conditions of abiotic stress to plants is commonly used as a treatment to augment plant defense responses against abiotic stress. This type of chemical priming is an example of *trans*-priming mentioned above. For example, priming Chinese crab apple (*Malus hupehensis*) with ABA helped to diminish the effects of cadmium (Cd) toxicity (Deng et al. 2022). Similarly, exogenous melatonin applied to tomato (*Solanum lycopersicum*) under salt stress increased its salt tolerance by several mechanisms, including the regulation of enzymes involved in proline and carbohydrate metabolism in seedlings (Siddiqui et al. 2019). Exogenous spermidine alleviated the detrimental effects of heat stress in *O. sativa* ssp. *japonica* varieties Wuyunjing 24 and Ningjing 3 (Tang et al. 2018), and exogenous menadione sodium bisulfite strengthened the response of bread wheat (*T. aestivum*) against alkaline stress (Jiménez-Arias et al. 2019).

Exogenous chemical application can specifically regulate gene expression to promote stress tolerance, as was observed in tomato (*S. lycopersicum*) plants facing low light stress. Exogenous GR24 application significantly upregulated the expression of PSII genes, such as *psbA* and *psaB*, helping to maintain photosynthetic efficiency (Lu et al. 2019). Likewise, exogenous SA application improved the defense of alfalfa (*M. sativa*) against freezing stress by inducing the expression of specific genes that led to enhanced antioxidant enzyme production (Wang et al. 2023).

Both lncRNAs and exogenous chemical applications may play a role in plant stress response; therefore, the link between them is intriguing. Gene expression regulated by lncRNA-mediated pathways is a possible mechanism by which exogenous chemical application leads to stress tolerance.

**Fig. 5:**
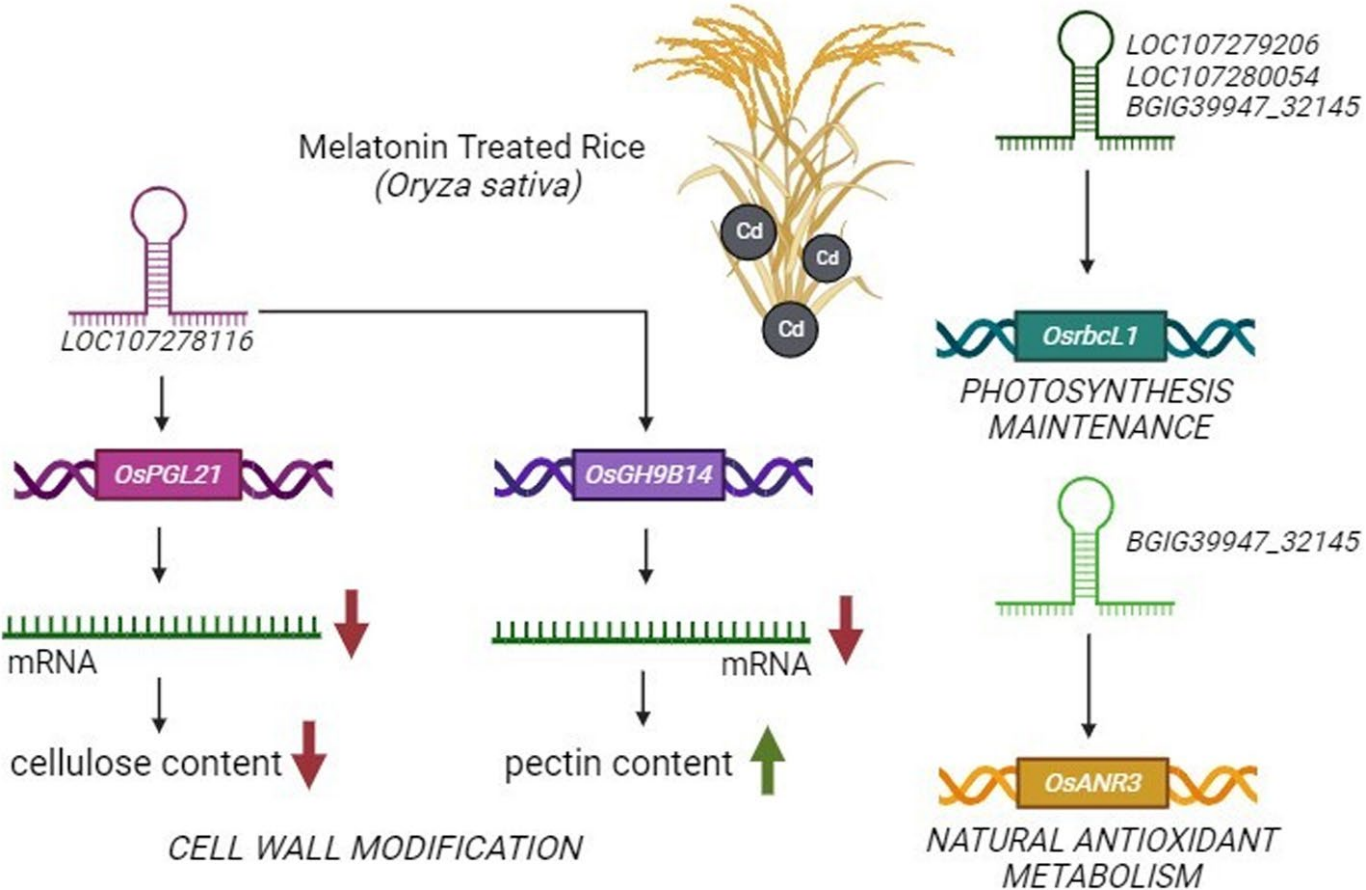
Melatonin enhances cadmium stress tolerance in rice (*O. sativa*) by lncRNA-mediated pathways. 125 lncRNAs were differentially expressed in chemically treated plants. These RNAs regulate genes that are involved in cell wall modifications to increase cadmium sequestration, maintain photosynthesis by protecting chloroplasts, and reduce oxidative damage by synthesizing antioxidants

Rice (*O. sativa*) plants exposed to significant Cd concentrations exhibited signs of heavy metal toxicity and were treated with melatonin. Melatonin is known to alleviate these adverse effects, especially by counteracting oxidative stress and altering Cd uptake and sequestration. About 125 lncRNAs were differentially expressed in plants in this experiment. A notably larger number of lncRNAs were expressed in plants exposed to both Cd and melatonin than in plants treated with either Cd or melatonin alone. These lncRNAs were involved in modifications of the plant cell wall by regulating the expression of genes that increased pectin content and decreased cellulose content, allowing Cd to be immobilized within the cell wall itself. In addition, lncRNAs play a role in preserving the integrity of chloroplasts by targeting genes associated with the metabolism of natural antioxidants (Qiu et al. 2024) (Fig. 5).

In poplar (*Populus* × *euramericana*) leaves treated with SA, 49 lncRNAs related to the stress response were differentially expressed. The target genes of these lncRNAs were mainly involved in MAPK signaling (important for regulating plant processes), secondary metabolism, and hormone signal transduction. One such gene, *cytokinin dehydrogenase 1* (target of lncRNA *MSTRG.27124.2*), was thought to play a role in drought stress tolerance. Another gene, *fructose-diphosphate aldolase 1*, was targeted by seven lncRNAs (*MSTRG.18764.2*, *MSTRG.24214.3*, *MSTRG.27124.2*, *MSTRG.3816.2*, *MSTRG.3931.1*, *MSTRG.5940.1*, and *MSTRG.929.1*). Fructose-diphosphate aldolase activity was found to impact cold and salt stress tolerance (Zhang et al. 2023). Moreover, 412 lncRNAs were differentially expressed in strawberry (*Fragaria × ananassa*) as a response to exogenous ABA application, and these lncRNAs were predicted to be involved in pathways by which plants respond to heat, drought, and osmotic stresses (Chen et al. 2022) (Table 2).

Another study investigated how lncRNAs in cassava (*Manihot esculenta*) were affected by treatment with polyethylene glycol (PEG) and melatonin, which induced drought stress and tolerance to drought stress, respectively. Differentially expressed lncRNAs included 75 under PEG treatment, 68 under melatonin treatment, and 42 under both treatments. In addition, 28 lncRNA-mRNA pairs involved in the regulation of neighboring genes were identified. These lncRNAs were involved in light signaling, fatty acid synthesis and elongation, secondary metabolism, and tetrapyrrole synthesis (Ding et al. 2019) (Table 3).

**Table 3:** Studies on the effect of chemical treatments on lncRNAs

**Plant Species**	**Chemical Applied**	**No. of Differentially Expressed** **LncRNAs**	**Target Genes**	**Stress Tolerance Enhanced by Treatment**	**Reference**
*Populus × euramericana* (poplar)	salicylic acid	49	*CKX1* *FBA1*	drought	(Zhang et al. 2023)
				cold	
				salt	
*Fragaria × ananassa* (strawberry)	ABA	412	-	heat	(Chen et al. 2022)
				drought	
				osmotic	
*Manihot esculenta* (cassava)	melatonin	68	-	drought	(Ding et al. 2019)

Some researchers have studied the activity of specific lncRNAs as a result of chemical treatments. A study showed that when *Arabidopsis* was treated with ABA, the amount of the lncRNA *TE-lincRNA11195* significantly increased. *TE-lincRNA11195* expression also varied considerably under salt, heat, cold, and drought stresses, suggesting that it plays a role in abiotic stress response (Wang et al. 2017). A similar study showed that the lncRNA *DRIR* was strongly induced by drought and salt stresses in *Arabidopsis* treated with ABA. *DRIR* appeared to play a role in regulating the expression of genes involved in drought and salt stress tolerance, such as *P5CS1* and *RD29A* (Qin et al. 2017) (Table 3).

The marneral cluster of *Arabidopsis* contains the *AT5G00580* gene, which is transcribed into the *MARS* lncRNA. *MARS* is most strongly induced in response to heat stress and exogenous ABA application. The expression of other genes (such as those that influence seed germination) located in the marneral cluster is regulated by the formation of a chromatin loop between the *MRN1* locus and a distal enhancer element. MARS overaccumulation leads to chromatin remodeling because of its interactions with LIKE HETEROCHROMATIN PROTEIN 1, which facilitates the process (Roulé et al. 2022) (Table 4).

**Table 4:** Studies on specific lncRNAs in *Arabidopsis* induced by chemical treatment

**Plant Species**	**Chemical Applied**	**Induced LncRNA**	**Target Genes**	**Stress Tolerance Enhanced by Treatment**	**Reference**
*Arabidopsis thaliana*	ABA	*TE-lincRNA11195*	-	drought	(Wang et al. 2017)
				cold	
				salt	
		*DRIR*	*P5CS1 RD29A*	heat	(Qin et al. 2017)
				drought	
				osmotic	
		*MARS*	*MRN1*	drought	(Roulé et al. 2022)

However, there are studies that provide evidence contrary to this link. For example, a study of drought stress in cassava (*M. esculenta*) found that the major lncRNA *DIR*, predicted to enhance the drought tolerance response, was not significantly affected by ABA or jasmonic acid treatment (Dong et al. 2022).

There is limited research on the effects of exogenous applications of chemicals on lncRNAs in plants, especially those with horticultural value, and further investigation is required.

## Limitations

According to the selected effects theory of function, the function of a trait is the function for which the trait was naturally selected; thus, a true function should have an evolutionary context (Neander 1991). Because most lncRNAs tend to show low sequence conservation, proving functionality is a difficult task (Sang et al. 2021). Most of the recent studies discussed in this paper proposed that an increased expression of lncRNAs under abiotic stress can indicate their roles in stress tolerance. In these experiments, Gene Ontology (GO) and Kyoto Encyclopedia of Genes and Genomes (KEGG) enrichment analyses were performed to determine the biological processes, cellular locations, and molecular functions impacted by the stress condition and the pathways involving lncRNAs. Despite being a good starting point to identify novel lncRNAs, studies of this nature cannot definitively prove that the relationship between lncRNAs and abiotic stress is not simply correlational. Other studies were able to identify how lncRNAs were involved in certain molecular mechanisms, but these were still causal role functions (Graur et al. 2013).

Some studies involved transgenic lines of plants that were edited with CRISPR to knock out or overexpress certain lncRNAs. This allowed for the observation of phenotype changes, which is more conclusive proof of lncRNA functionality (Wierzbicki et al. 2021). Therefore, more research of this nature is required in the future. A limited number of studied plant lncRNAs have irrefutable biological functions with well-understood molecular mechanisms. As research in this field continues, attempts must be made to establish definitive functions of other lncRNAs as well. An important step is designing biochemical experiments that do not extensively depend on computational tools to help gather evidence that could corroborate the predictions of many published studies. In terms of chemically induced abiotic stress tolerance, the small body of literature is unable to identify if the effect of the priming stimulus is lncRNA mediated or if stress tolerance is a result of other mechanisms. Nevertheless, the increased expression of lncRNAs in such conditions is compelling and worth researching.

## Conclusions

Growing evidence over the past decade suggests that the bulk of the eukaryotic genome is transcribed, a phenomenon known as “pervasive transcription”, resulting in increasing research on the possible biological roles and functional mechanisms of ncRNAs, including lncRNAs. However, it is often unclear whether a lncRNA in a specific condition exerts a function or if it is just a byproduct of other noisy transcriptional processes (Jensen et al. 2013). The overall findings of this review suggest that lncRNAs coordinate gene expression in response to various environmental stimuli that have negative effects on plants. These stimuli include stresses such as heat, drought, cold, and salt. While several mechanisms of action of lncRNAs have been identified thus far, most studies focus on the identification of lncRNAs rather than their functional characterization. The latter is often only predicted computationally, and it remains a difficult endeavor to prove such mechanisms by biochemical or mechanistic studies. Thus, further research is required (Manavella et al. 2023). There is mounting evidence suggesting that the abiotic stresses discussed in this review will become more prevalent in the coming years as climate change worsens. Understanding the many regulatory mechanisms, including those involving lncRNAs, which control and promote adaptive responses to stress in different plant species is important for finding ways to maintain plant productivity and produce horticultural crop species with reduced susceptibility to stress.

## Data Availability

Not applicable.

## Abbreviations

ABA: Abscisic acid
*ABA2*: *Abscisic acid-deficient 2*
*ALT TAS3*: *Trans-acting small interference RNA3*
*APOLO*: *AUXIN-REGULATED PROMOTER LOOP*
*APX1*: *Ascorbate peroxidase 1*
*ceRNA*: *Competing endogenous RNA*
*COLDAIR*: *COLD-ASSISTED INTRONIC NON-CODING RNA*
*COLDWRAP*: *COLD OF WINTER-INDUCED NON-CODING RNA FROM THE PROMOTER*
*COOLAIR*: *Cold-induced long antisense intragenic RNA*
*ELENA1*: *ELF-18 induced lncRNA1*
*FLAIL*: *Flowering-associated intergenic lncRNA*
*FLC*: *FLOWERING LOCUS C*
HeFP: Helicase family protein
HSP: Heat-shock proteins
IAA: Indole-3-acetic acid
lincRNA: Long intergenic lncRNA
lncRNA: Long non-coding RNA
*MARS*: *MARneral Silencing lncRNA*
miRNA: microRNA
ncRNA: Non-coding RNA
OST1: Open stomata 1
PEG: Polyethylene glycol
*PR1*: *PATHOGENESIS-RELATED GENE 1*
*PRX52*: *Peroxidase 52*
ROS: Reactive oxygen species
rRNA: Ribosomal RNA
siRNA: Small interfering RNA
SOD: Superoxide dismutase
sRNA: Small RNA
TF: Transcription factor
